# Keratinocyte self-renewal and differentiation is dictated by extrinsic signals from dermal extracellular matrices

**DOI:** 10.1098/rsob.240417

**Published:** 2025-10-22

**Authors:** Chee Wai Wong, Catherine F. LeGrand, Beverley F. Kinnear, Simon L. I. J. Denil, Danielle E. Dye, Paula Benny, Jerry K. Y. Chan, E. Birgitte Lane, Deirdre R. Coombe

**Affiliations:** ^1^ Curtin Medical School, Curtin University, Perth, Western Australia, Australia; ^2^ Curtin Medical Research Institute, Curtin University, Bentley, Western Australia 6102, Australia; ^3^ Institute of Medical Biology, Agency for Science, Technology and Research (A*STAR) 138648, Singapore; ^4^ Obstetrics and Gynaecology Academic Clinical Program, Duke-NUS Medical School 169857, Singapore; ^5^ Department of Reproductive Medicine, KK Women’s and Children’s Hospital 229899, Singapore; ^6^ Department of Obstetrics and Gynaecology, Yong Loo Lin School of Medicine 119228, Singapore; ^7^ A*STAR Skin Research Laboratories (ASRL), Agency for Science, Technology and Research (A*STAR) 138648, Singapore

**Keywords:** skin development, wound healing, extracellular matrix, adult dermal matrix, fetal dermal matrix, keratinocyte differentiation, keratinocyte self-renewal

## Abstract

Fetal skin has an intrinsic regenerative capability to restore an injured site’s architecture and functionality. This is preserved until the third trimester, when wound healing transitions to a scarring reparative response. This change coincides with the dynamic remodelling of dermal extracellular matrix (ECM). Here, we used primary human fetal or adult dermal fibroblast (fHDF and aHDF)-derived ECMs to demonstrate that different extrinsic signals from these ECMs dramatically altered gene expression in a primary human keratinocyte population grown on these matrices. Gene array data revealed keratinocytes grown on fHDF ECM markedly upregulated expression of cell-cycle genes, whereas on aHDF ECM expression of differentiation genes was favoured. Detailed proteomic analyses indicated compositionally distinct ECMs were deposited by aHDFs and fHDFs. Moreover, aHDFs and fHDFs contained subpopulation(s) that differentially expressed CD90, CD146 and CD26. On fHDFs the extracellular domain of CD26 was shed whereas on aHDFs full-length CD26 dominated. The proteomic and gene array data supported the fine-tuning of BMP/TGFβ/SMAD signalling pathways being a mechanism by which fetal matrices promote keratinocyte self-renewal. Collectively, these findings revealed that a fundamental aspect of skin development is dictated by the ECM of the dermis, specifically extrinsic signals from dermal fibroblast ECM direct keratinocyte self-renewal or differentiation.

**Figure:**
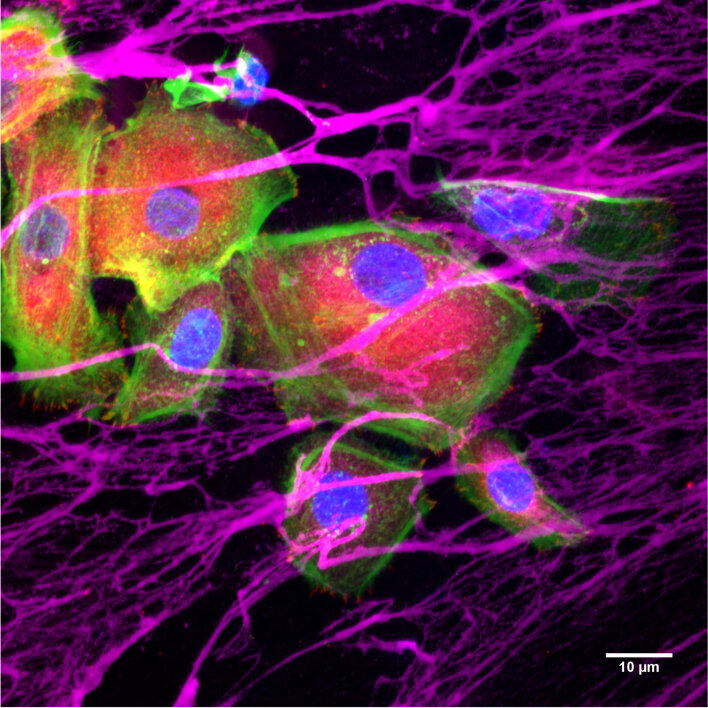

## 1. Introduction

Skin is a complex organ comprising an outer epidermis, an underlying dermis and appendages. The continual maintenance of the epidermis is essential for skin’s function as a protective barrier [1]. During embryogenesis, epidermal development is initiated when surface ectoderm commits to an epidermal fate, giving rise to a layer of basal keratinocytes [2]. At this early stage, basal keratinocytes are programmed for active proliferation [1]. But, upon receiving developmental cues, they become committed towards stratification and form the distinct layers of differentiated cells seen in adult epidermis. The structural integrity of the epidermis is maintained by the homeostatic regulation of proliferation and differentiation of basal keratinocytes [3]. The transition in developmental programming of the fetal to the adult epidermis coincides with changes in skin wound healing, from regenerative healing to a scarring, reparative response [3], suggesting that they could be intrinsically linked.

During epidermal development, the extracellular matrix (ECM) of the dermis changes. While the principal ECM components within both fetal and adult dermis are collagen I and collagen III, the ECM of the fetal dermis has a higher ratio of collagen III to collagen I [4]. By the third trimester of fetal development, the ratio changes and collagen I becomes predominant. This shift correlates with the change from regenerative healing to scarring repair [5]. A feature of the fetal dermal ECM is the abundance of glycosaminoglycans (GAGs), specifically chondroitin sulfate [6] and hyaluronan [7]. These GAGs play essential roles in ECM structure and they influence cell behaviours such as proliferation and migration [8,9]. ECM glycoproteins like fibronectin [6] and proteoglycans such as decorin [10] and versican [11] are also differentially expressed in adult and fetal dermal ECMs. Such differences in ECM composition may provide signals to direct the transition of keratinocyte programming from regeneration to repair and differentiation. Since fibroblasts are the major contributors to dermal ECM, insights into differences between the ECM proteins secreted by adult and fetal dermal fibroblasts should assist our understanding of how ECM contributes to the different behaviours of adult and fetal keratinocytes seen *in vivo*.

The dermis contains spatially distinct fibroblast subpopulations that function differently to support normal homeostasis and wound healing, and these subpopulations contribute to different healing outcomes [12]. Several distinct fibroblast subpopulations were identified in murine adult dermis through spatial and single-cell transcriptional profiling [13]. These subpopulations are phenotypically unique in the composition of the ECM that they secrete [13]. Other studies revealed that fibroblasts which expressed engrailed 1 (EN1) prior to differentiation, but subsequently expressed dipeptidyl peptidase IV (CD26/DPP4), were responsible for scar formation during wound healing in mouse models [14]. In the human dermis, CD26 expression is age dependent, with very little CD26 being expressed by fetal and neonatal fibroblasts [15], but adult fibroblasts express this marker [15]. CD26^+^ fibroblasts were pro-fibrotic and expressed more fibronectin than CD26-knockout fibroblasts [15]. A better understanding of fibroblast subpopulations within human dermis and the ECM proteins they secrete will indicate the extent to which conclusions from murine systems also apply to the human situation.

*In vivo* dermal fibroblasts exist in a microenvironment that is densely packed with large macromolecules of a highly organized dermal ECM. However, to study in detail how the composition of the ECM secreted by dermal fibroblasts differs in fetal and adult skin and how these differences may alter keratinocyte behaviour necessitates an *in vitro* culture system. In a typical *in vitro* culture system cells are maintained in a dilute macromolecular solution (1–10 mg ml^−1^) which is considerably more dilute than the extracellular fluid cells encountered *in vivo* (ranging from 20.6–80 mg ml^−1^) [16]. In such a dilute environment cells lose some of their *in vivo* functionality resulting in less secretion of ECM components and a reduction in the molecular interactions that are required for proper ECM assembly. In recent years, we and others have used a process of ‘macromolecular crowding’, whereby large inert macromolecules are added to the culture medium, to better replicate a tissue-like macromolecular density [17–19]. We used a mixture of Ficoll 70 and Ficoll 400, Ficoll being a large neutral hydrophilic polysaccharide which occupies space without interacting with, or removing from, the culture medium biological molecules the cells require for optimum performance [17]. Under these conditions human dermal fibroblasts produced a superior ECM that was structurally well organized, but most importantly the fibroblasts did not differentiate into myofibroblasts and the ECM supported keratinocyte expansion. Others similarly reported a lack of myofibroblast differentiation [20] and macromolecular crowded cell cultures are now preferred for regenerative medicine applications and tissue engineering [20–24].

Previously, we revealed that an ECM deposited by human dermal fibroblasts is superior to either a substrate of collagen I [17] or an ECM from murine 3T3 fibroblasts [25] for supporting the proliferation of primary human keratinocytes. Here, we further developed this work by investigating the differences in the phenotype of human adult and fetal dermal fibroblasts (aHDFs and fHDFs), and the ECM proteins they deposit *in vitro*. We also examined whether ECM deposited by aHDFs and fHDFs differentially influenced the behaviour of primary human keratinocytes. Lastly, we explored whether fibroblast subpopulations were present in the aHDF and fHDF populations used in our study. We found that our aHDFs differed from the fHDFs in their morphology. Quantitative proteomic analysis revealed differences in the composition of the ECM deposited by aHDF and fHDF. Importantly, we detected marked differences in the gene expression profiles of primary keratinocytes following their growth on matrices deposited by either aHDFs or fHDFs. Keratinocytes grown on fHDF matrices showed upregulated expression of cell cycle genes, whereas the same keratinocytes grown on aHDF matrices had upregulated expression of a combination of cell cycle and differentiation genes. These data indicate that the ECM deposited by either aHDFs or fHDFs contain distinct signals that can promote differentiation, or self-renewal in keratinocytes, respectively. The likely involvement of BMP/TGFβ/SMAD signalling in these processes is discussed.

## 2. Results

### 2.1. fHDFs are phenotypically different from aHDFs

**Figure 1.**
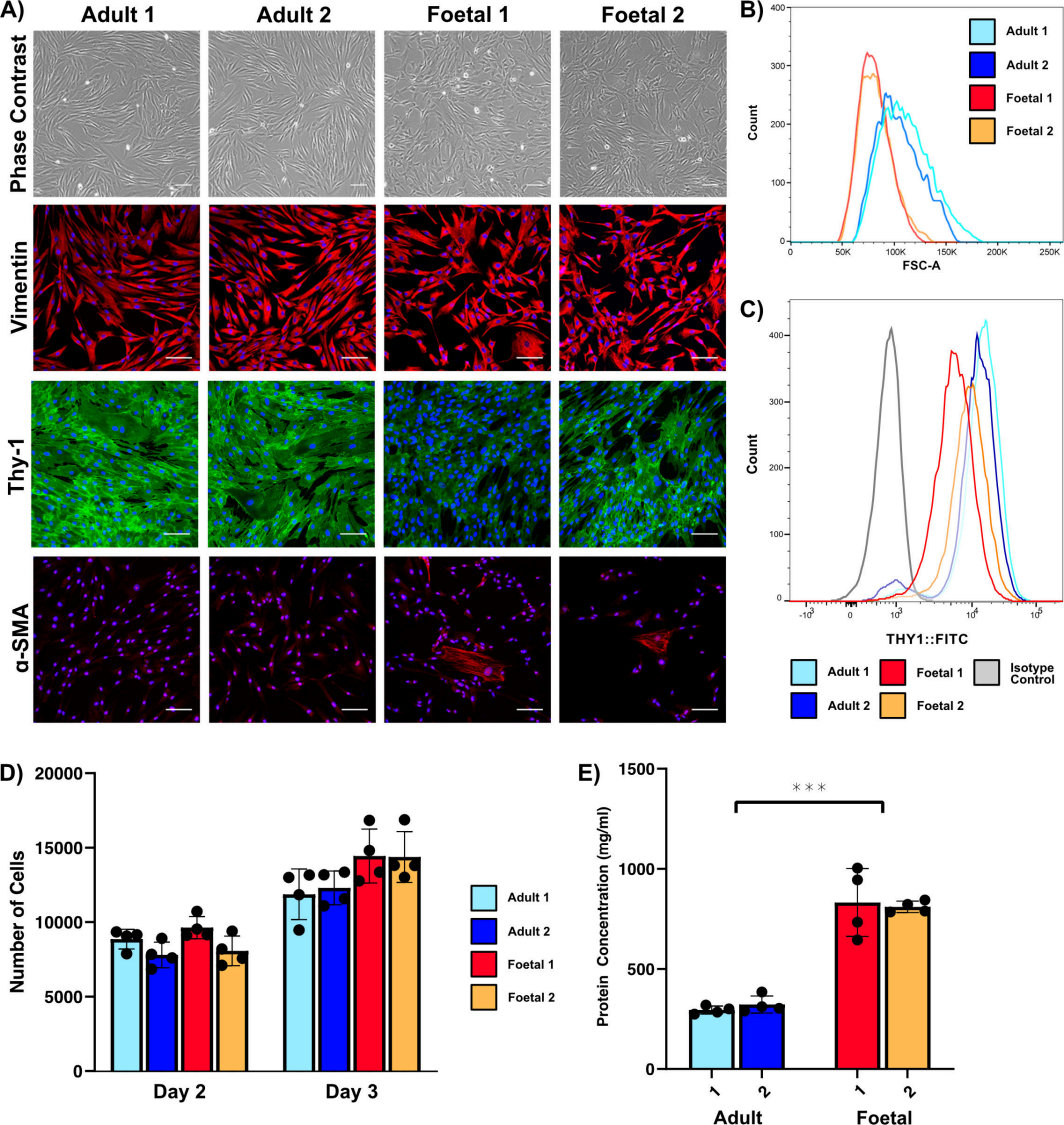
Characterization of adult- and fetal- human dermal fibroblasts. (A) Phase contrast images show the morphology of aHDFs and fHDFs. The fibroblasts were also stained with antibodies recognizing vimentin, Thy-1 or alpha-smooth muscle actin (α-SMA). The secondary antibody was an Alexa Fluor 546-conjugated or Alexa Fluor 488-conjugated antibody. Nuclei were stained with DAPI (blue). Scale bars are 100 µm. (B) Histogram showing cell size (FSC-Area) of aHDFs and fHDFs determined from FACS. (C) Histogram of fluorescence-activated cell sorting (FACS) analysis of Thy-1 on aHDFs and fHDFs. (D) Proliferation of aHDFs and fHDFs. The number of fibroblasts after culturing for 2 or 3 days. Data are expressed as mean ± SD. Shown is a representative of triplicate experiments. (E) Quantification of ECM deposited by aHDFs and fHDFs after decellularization. Data are expressed as mean ± SD. The figure is representative of three experiments. ***p < 0.001.

Dermal fibroblasts were derived from two adult and two fetal skin samples. Both fHDFs came from samples acquired within the first and second trimester of pregnancy, when fetal skin regeneration is known to occur. Phase-contrast microscopy revealed that aHDFs have a uniform, thick, spindle-like morphology, which is a typical fibroblast phenotype, whereas fHDFs have a more variable, often stellate morphology (figure 1A). In general, fHDFs appeared smaller than aHDFs, an observation confirmed by flow cytometric analysis using the forward scatter area parameter (figure 1B). While considerable overlap in cell sizes occurred in all samples, the median size of cells from both fHDF samples is to the left of the median size of cells from both aHDF samples, indicating most of the fHDFs were smaller than the majority of aHDFs. Both aHDFs and fHDFs stained positively for the fibroblast markers Thy-1 (CD90) and vimentin. Surprisingly, Thy-1 staining intensity was lower in fHDFs compared with that seen in the aHDFs (figure 1A,C), and flow cytometry analyses revealed a small but distinct subpopulation of aHDFs from both samples failed to stain above the level of the negative control with the anti-Thy-1 mAb (figure 1C). The possibility of a subpopulation of myofibroblasts within the aHDF and fHDF populations was investigated. To distinguish myofibroblasts, an antibody recognizing alpha-smooth muscle actin (α-SMA) was used to reveal prominent α-SMA stress fibres, which are a distinguishing feature of myofibroblasts. In the absence of macromolecular crowding (MMC) only a small subpopulation of myofibroblasts was detected within the fHDFs, and no myofibroblasts were seen within the aHDFs (figure 1A).

Brink *et al.* [26] reported more rapid growth and greater production of ECM by fetal rat dermal fibroblasts compared with similarly cultured adult fibroblasts. To investigate whether this was true for our fibroblast populations, cell proliferation rates and quantification of the total ECM protein produced by aHDFs and fHDFs were determined. There was no difference in the numbers of fHDFs and aHDFs on day 2 of culture, but more fHDFs than aHDFs were recorded on day 3 of culture, although this was not statistically significant (figure 1D). The relative amounts of ECM protein produced by the fibroblasts was determined following 6 days in culture under MMC conditions and after decellularization [17]. The fHDFs deposited approximately double the quantity of protein compared with the aHDFs (figure 1E).

### 2.2. ECM from aHDFs and fHDFs are compositionally distinct

**Figure 2.**
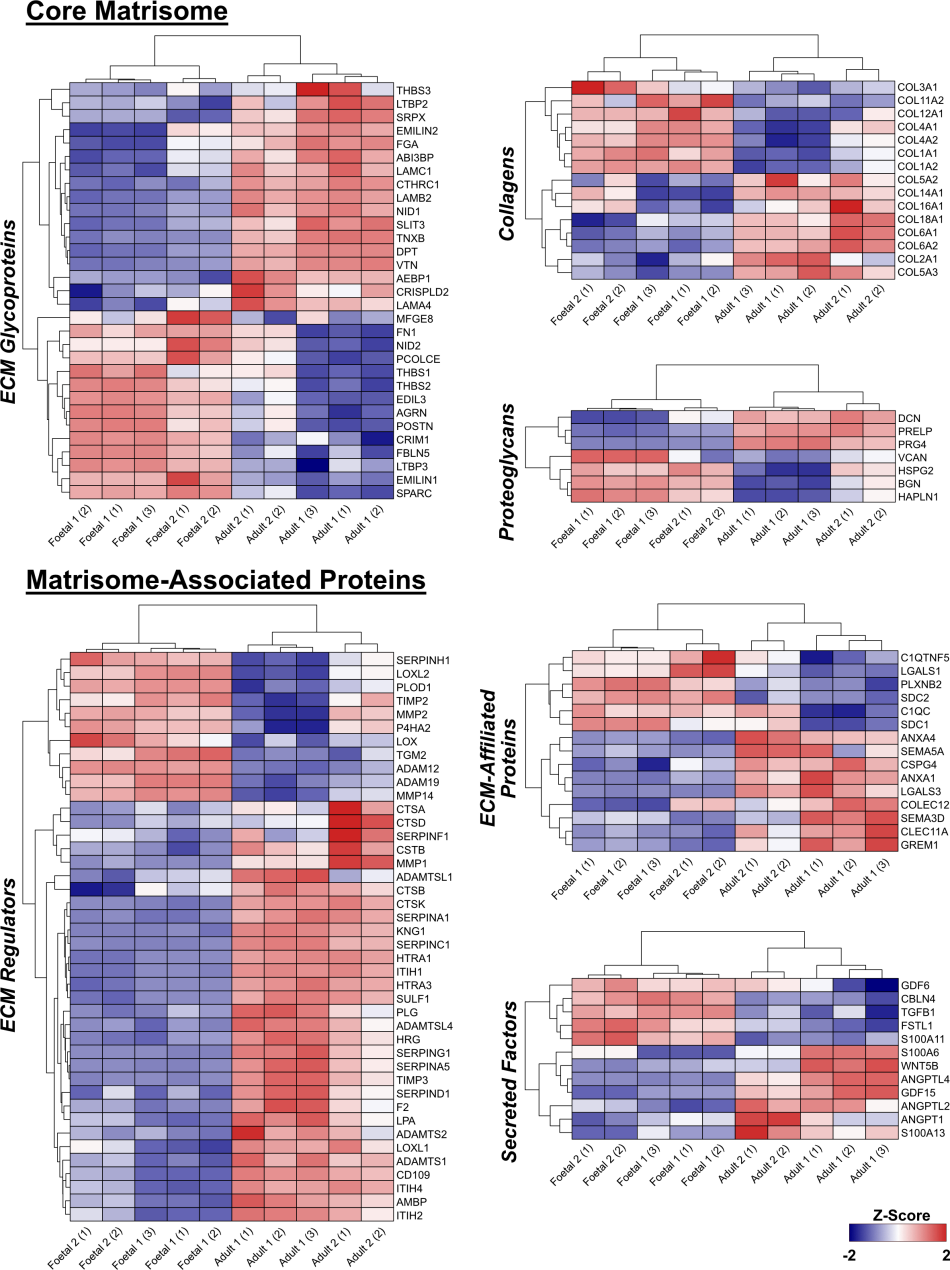
Comparison of matrisome composition between adult and fetal human dermal fibroblasts. Core matrisome protein and matrisome-associated protein compositions of the acellular ECM generated from aHDFs and fHDFs were compared and shown as a heatmap. The core matrisome proteins are subdivided into ECM glycoproteins, collagens and proteoglycans. The matrisome-associated proteins are subdivided into ECM-affiliated proteins, ECM regulators and secreted factors. Replicates for each sample are: Foetal 1, *n* = 3; Foetal 2, *n* = 2; Adult 1, *n* = 3; Adult 2, *n* = 2.

The composition of the ECM secreted by aHDFs and fHDFs was investigated using quantitative proteomics. Fibroblasts were cultured for 7 days under MMC conditions before decellularization and solubilization of the matrix proteins was performed for mass spectrometry analyses. The resultant proteomic dataset was curated for ECM proteins using the human matrisome database (Matrisome DB), which categorizes ECM proteins into ‘core matrisome’ (glycoproteins, collagens and proteoglycans) and ‘matrisome-associated proteins’ (ECM-affiliated proteins, ECM regulators and secreted factors) [27]. Figure 2 shows the quantitative heat-maps resulting from these analyses. Despite being derived from different individuals, each aHDF sample produced ECMs with a similar protein composition, as did the two fHDF samples. However, there were marked differences in the abundance of many proteins between the aHDF and fHDF ECMs.

Among the core matrisome proteins detected were the basement membrane glycoproteins, laminin chains: LAMA4, LAMB2 and LAMC1; collagen IV, nidogens: NID1 and NID2, and HSPG2 (perlecan). Higher levels of the laminin chains and less HSPG2 and collagen IV were detected within the aHDF ECMs. Interestingly, aHDF and fHDF ECMs were enriched respectively for NID1 or NID2. Of the connective tissue ECM components, collagens I and III were less abundant in aHDF ECMs compared with the ECMs from fHDFs, while the reverse was true for several other collagen chains. Other ECM glycoproteins detected in different quantities between the ECMs were VTN (vitronectin), which was higher in the aHDF ECM, and FN1 (fibronectin), which was markedly less abundant in one adult ECM (Adult 1), but the difference between its abundance in the ECMs of Adult 2 and the fHDF ECMs was less clear. Proteins involved in collagen fibrillogenesis, like AEBP1 (adipocyte enhancer-binding protein 1) and DPT (dermatopontin), and in elastin fibre formation, like FBLN5 (fibulin 5), EMILIN1 and EMILIN2 (elastin-microfibril-interface-located-proteins 1 and 2) were present in different quantities: DPT, AEBP1 and EMILIN2 were more abundant in adult matrices but EMILIN1 and FBLN5 were more abundant in fetal matrices. DPT also interacts with the proteoglycan DCN (decorin), which was also abundant in the adult ECMs. AGRN (agrin), POSTN (periostin), SPARC (secreted protein acidic and rich in cysteine) and CRIM1 (cysteine-rich motor neuron 1) were all higher in the fHDF ECMs

A multitude of ECM modifiers and inhibitors was apparent in aHDF ECM. These included ADAMTS 1 and 2, (a disintegrin and metalloproteinase with thrombospondin motifs 1, 2), TIMP3 (tissue inhibitor of metalloproteinases 3), AMBP (alpha-1-microglobulin/bikunin precursor), CD109, F2 (coagulation factor II, thrombin), HTRA1, HTRA3 (high-temperature requirement A serine peptidase 1, 3), ITIH1, ITIH2, ITIH4, (inter-alpha-trypsin inhibitor heavy chain 1, 2, 4), LOXL1 (lysyl oxidase like 1) and SERPIN (serine protease inhibitor) proteins. Whereas fetal ECMs appeared to have a higher proportion of matrix enzymes than inhibitors with MMP14 (matrix metalloproteinase-14), ADAM 12 and 19 (a disintegrin and metalloproteinase 12, 19), LOX (lysyl oxidase), LOXL2 and TGM2 (transglutaminase 2) being well represented. Three TGFβ (transforming growth factor beta) superfamily members were also detected: TGFβ1, GDF6 (growth differentiation factor 6) and GDF15 (growth differentiation factor 15). TGFβ1 was most abundant in the fHDF ECMs, and GDF15 was abundant in the aHDF ECMs. The abundance of GDF6 was low in the ECMs from one aHDF sample but not the other. Interestingly, the TGFβ1-inducible gene, FSTL1 (follistatin-like 1) [28], is most abundant in the fHDF ECM as are proteoglycans that bind TGFβ1, BGN (biglycan) and HSPG2 (perlecan). These, along with other ECM proteins and ECM-associated proteins (figure 2), denote differences in the composition of the ECMs deposited by aHDFs and fHDFs.

**Figure 3.**
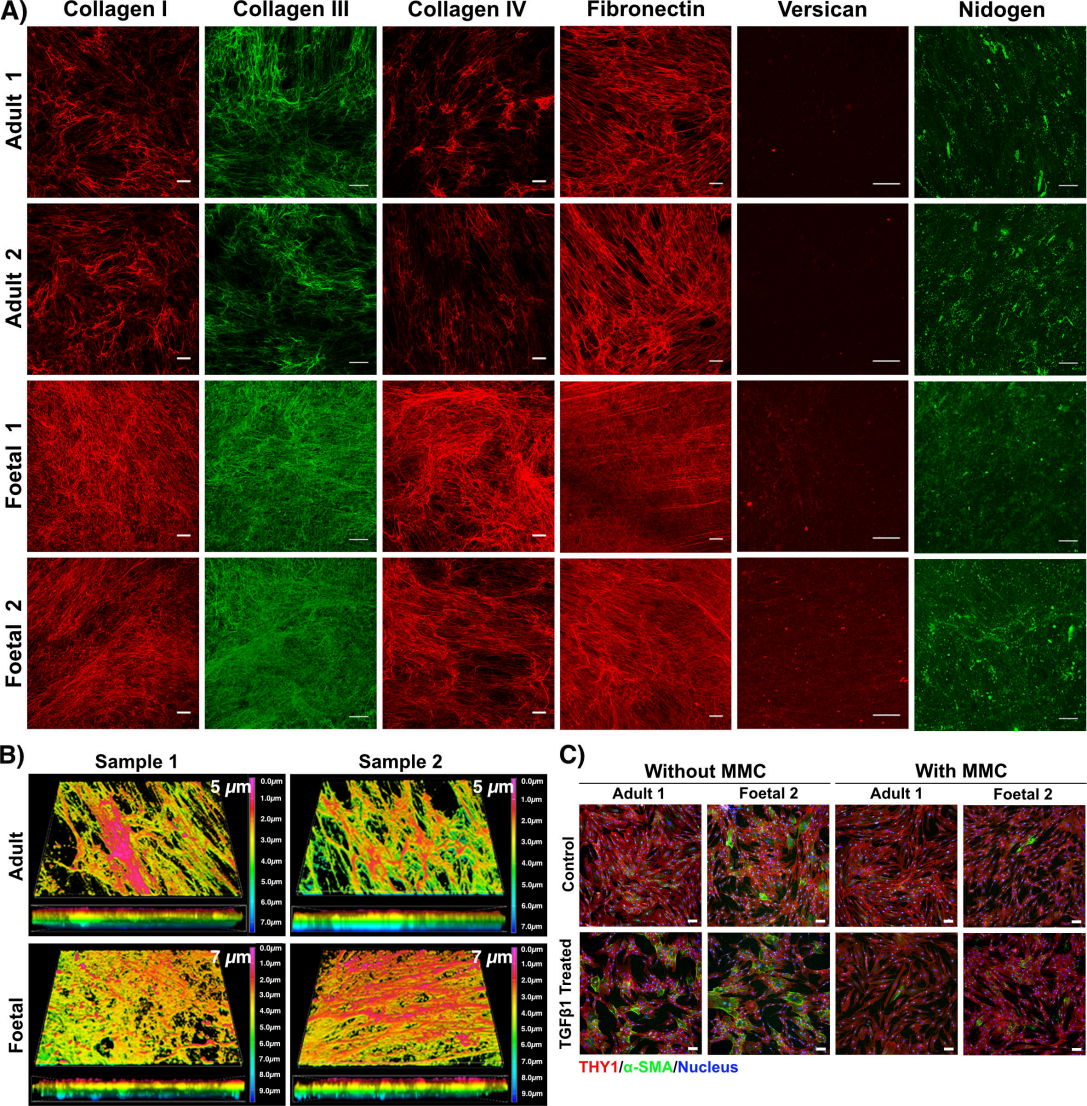
Characterization of ECM deposited by adult- and fetal- human dermal fibroblasts. (A) The acellular matrices generated by aHDFs and fHDFs were immunostained with antibodies recognizing collagen I, collagen III, collagen IV, fibronectin, versican or nidogen. Scale bars for collagen I, collagen III, collagen IV and fibronectin are 100 µm. Scale bars for versican and nidogen are 50 µm. (B) The thickness of the ECM deposited by aHDFs and fHDFs was measured after decellularization. ECMs were immunostained for collagen I. The colour coding represents the Z-depth location within the 3D z-stacked image. ECM thickness is indicated. (C) Myofibroblast status of aHDFs and fHDFs during ECM deposition with or without macromolecular crowding (MMC) and with or without TGFβ1 treatment. The cells were immunostained for α-SMA (green) and Thy-1 (red) and imaged by confocal microscopy. Nuclei were stained with DAPI (blue). Scale bars are 100 µm.

Immunofluorescence staining independently verified a selection of the proteomics results. The immunofluorescence analyses (figure 3A) were consistent with the quantitative proteomic data for the ECM proteins examined; they showed more collagens I, III and IV, and versican in the fHDF ECMs relative to the aHDF ECMs. However, differences in the abundance of fibronectin between the two ECM types were less clear. Similarly, detecting a difference in the staining levels between the fHDF and aHDF ECMs with the anti-nidogen antibody was difficult, possibly because the antibody used recognizes both NID1 and NID2 isoforms. Structural differences between the aHDF and fHDF ECM were noted. Fibronectin fibres and the fibres of collagens I, III and IV were thinner and more numerous in the fHDF matrices, giving rise to a more compact fibre meshwork in the fHDF matrix than the aHDF matrix.

To better visualize the arrangement of the ECM proteins deposited, a 3D z-stacked construct of the immunostained ECM layers at high magnification was generated using confocal microscopy (figure 3B; electronic supplementary material, figure S1). In the fHDF ECM, a fine reticular/basket-weaved pattern was observed for both collagens I and IV. By contrast, thick and dense parallel bundles of collagen I and collagen IV were seen in the aHDF ECM (electronic supplementary material, figure S1). The fHDFs produced a thicker ECM, as measured from the 3D z-stacked ECM constructs following staining for either collagen I or collagen IV (figure 3B; electronic supplementary material, figure S2). The increased fHDF ECM thickness corresponds with the increase deposition of ECM proteins by fHDFs (figure 1E).

Next, we examined whether myofibroblasts were present in these cell populations following culturing under MMC conditions. When cultured under MMC conditions, the number of myofibroblasts in both aHDF and fHDF cultures was reduced (figure 3C). This is consistent with our previous observation [17] and that of Ramalingam *et al.* [20]. It is known that TGFβ1 stimulation causes dermal fibroblasts to differentiate into myofibroblasts [29]. When our dermal fibroblast samples were cultured without MMC conditions and stimulated with TGFβ1 numerous cells in both adult and fetal samples stained with the α-SMA antibody, indicating myofibroblasts. However, when cultured under MMC conditions TGFβ1 stimulation did not result in myofibroblast differentiation in aHDF or fHDF samples (figure 3C).

### 2.3. fHDF and aHDF ECM differently impact keratinocyte behaviour

**Figure 4.**
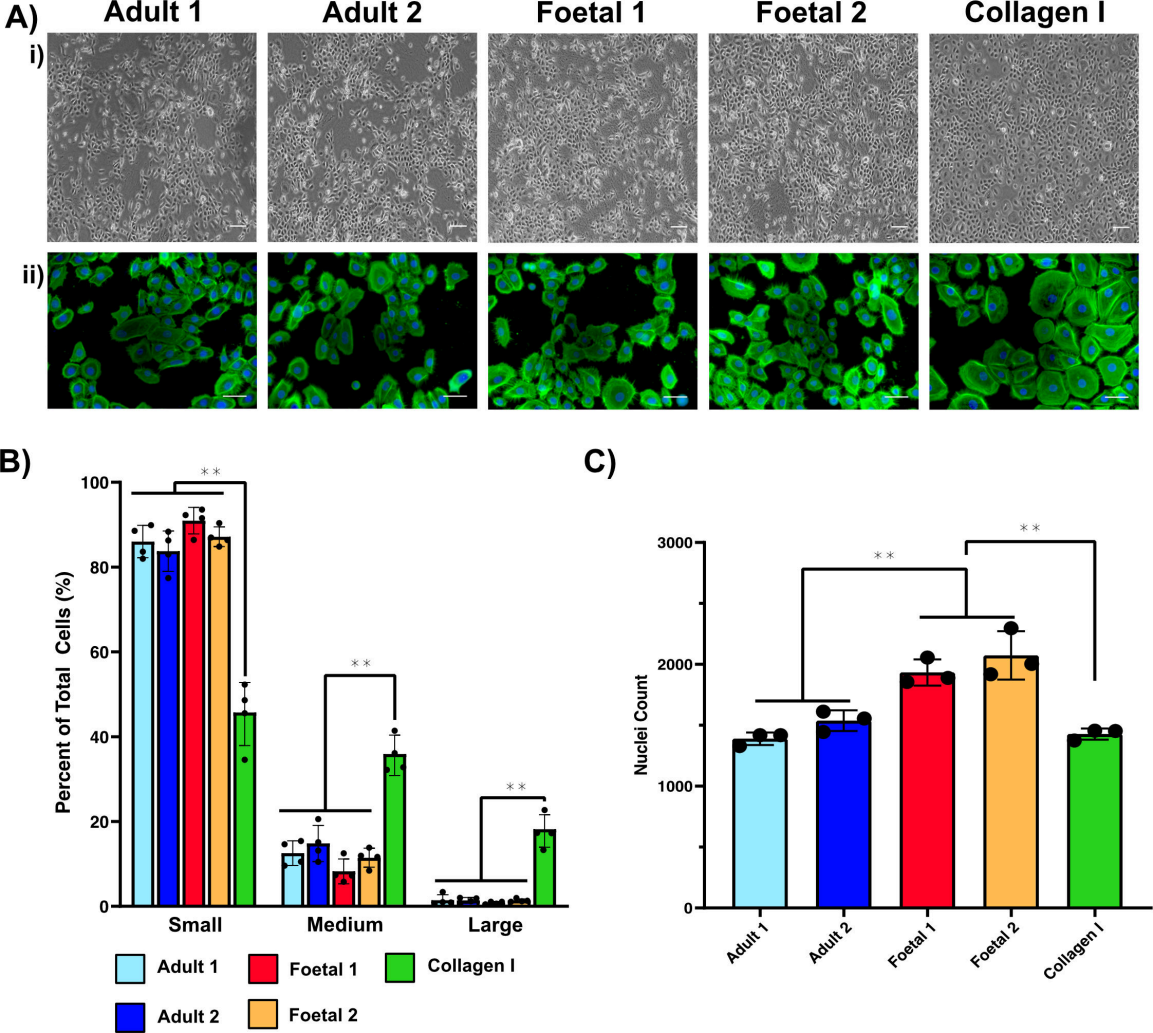
Characterization of keratinocytes cultured on collagen I, adult dermal fibroblast-derived ECM, or fetal dermal fibroblast-derived ECM. (A) Keratinocytes cultured on four complex ECMs secreted by fibroblasts derived from two adult samples and two fetal samples or on collagen I. (i) Phase contrast images of keratinocytes on the ECM and collagen I substrates. (ii) Representative images of Alexa-Fluor 488-phalloidin staining of F-actin in keratinocytes cultured on the substrates. Such images were used for cell area determination to define three size categories. (B) Keratinocyte size when grown on the substrates. The percentage of keratinocytes in each size category is shown. Cell size was categorized as small, medium or large based on cell area (see §5.10). Data are expressed as mean ± SD. Statistical analysis: ANOVA followed by a Tukey’s test. **p < 0.01. Shown is a representative of two experiments. (C) Keratinocyte numbers following growth on different substrates. Data are the mean nuclei count ± standard deviation. Shown is a representative of two experiments. Statistical analysis: ANOVA followed by a Tukey’s test. **p < 0.01.

The ability of aHDF and fHDF ECMs to support keratinocyte proliferation was investigated. Collagen I was used as the positive control, as this is the recommended substrate on which to culture keratinocytes when using defined keratinocyte serum-free medium [30] (DKSFM). The same population of primary human keratinocytes was seeded onto either aHDF ECMs, fHDF ECMs or collagen I and cultured for 3 days in DKSFM. On either aHDF or fHDF ECMs the keratinocyte population primarily comprised cells with a small, cobblestone morphology (figure 4A). However, when grown on collagen I, a number of large keratinocytes were present after the same time in culture, suggesting these keratinocytes had terminally differentiated [30]. Cell footprint size was visualized after staining with Phalloidin-Alexa 488 to demarcate the actin cytoskeleton. Cell footprint size was determined using the Cell Profiler software and assigned to one of three size categories, and the number of cells in each category calculated. A higher proportion of cells on collagen I were in the medium and large size categories (figure 4B) compared with cells on the cell-derived ECMs. Keratinocyte proliferation was assessed by determining the number of cell nuclei on each of the substrates after 3 days in culture. More nuclei were observed on the fHDF matrices, compared with the other substrates (figure 4C), indicating more proliferation occurred on the fHDF matrices.

### 2.4. The ECM type directs keratinocyte gene expression

**Figure 5.**
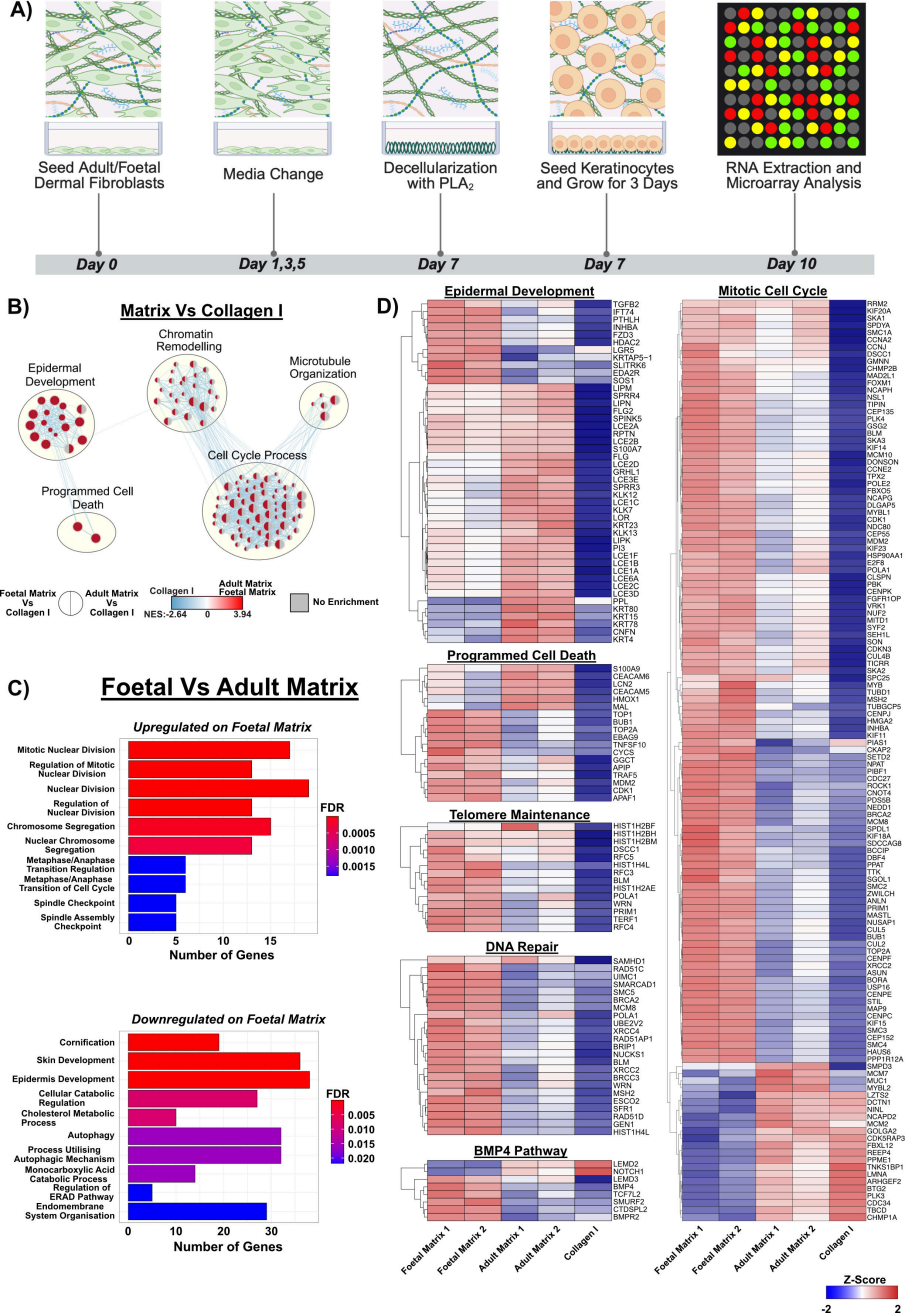
Gene expression analysis of keratinocytes grown on adult or fetal human dermal fibroblast ECMs using microarray. (A) Schematic diagram of the assay set-up, created in BioRender. (B) Enrichment map of genes expressed by keratinocytes grown on either collagen I, aHDF- or fHDFs-derived ECMs. Shown are GSEA results obtained when genes expressed by keratinocytes grown on aHDF- or fHDFs-derived matrices were compared with genes expressed by same keratinocyte population grown on collagen I. The results are separately displayed as pie charts. Red = enrichment in genes transcribed by keratinocytes grown on aHDF- or fHDFs-derived matrices. Blue = enrichment in genes transcribed by keratinocytes on collagen I. Colour intensity is indicative of the enrichment significance of a particular gene set and the circle sizes vary according to the number of genes. (C) ClusterProfiler GO enrichment results of keratinocytes grown on fHDF-derived ECMs versus keratinocytes grown on aHDF-derived ECMs. FDR: False discovery rate. (D) Transcriptome heatmap profiles of genes transcribed by keratinocytes grown on the different ECMs and collagen I. Categorization is according to epidermal development, programmed cell death, DNA repair, telomere maintenance, mitotic cell cycle and the BMP4 pathway.

To investigate whether aHDF and fHDF ECMs differently influenced keratinocyte gene expression a microarray analysis was performed. As before, collagen I coated tissue culture plastic was the control. Keratinocytes were cultured on the three substrates, in DKSFM, for 3 days before total RNA was extracted for microarray analysis using the Affymetrix HuGene 2.0 ST Chipset (figure 5A). Unsupervised hierarchical clustering analysis revealed that samples clustered according to the substrate on which the keratinocytes were grown (electronic supplementary material, figure S3).

Initially, differences in the genes expressed by keratinocytes on HDF ECMs and collagen I were investigated using gene set enrichment analysis (GSEA). The genetic pathways enriched in the keratinocytes on the different substrates, were displayed as a single, integrated enrichment map, generated using Cytoscape [31]. This map indicated that both aHDF and fHDF ECMs elicited genetic signalling pathways involving epidermal development and programmed cell death (figure 5B). However, only keratinocytes grown on fHDF matrices showed enrichment for genes involving chromatin remodelling, microtubule organization or cell cycle. When the keratinocytes grown on aHDF or fHDF ECMs were compared, it was clear that cell cycle related genes, particularly those involved in mitotic nuclear division and chromosome segregation, were highly upregulated in keratinocytes cultured on an fHDF ECM (figure 5C). Whereas genes involved in cornification and skin and epidermal development were downregulated in keratinocytes on fHDF ECMs. Our transcriptomic heat-map profile revealed that while keratinocytes cultured on both aHDF and fHDF ECMs were enriched for some epidermal development and programmed cell death genes, the subset of genes that were highly expressed differed according to the matrix type upon which the keratinocytes were grown (figure 5D). Genes indicating terminal differentiation of keratinocytes such as *LCE1A*, *LCE1B* [32] (late cornified envelope protein 1A, 1B), *KLK12* and *KLK13* [33] (kallikrein related peptidase 12, 13) were only highly expressed in keratinocytes from aHDF ECMs (figure 5D). By contrast, genes associated with cell cycle, DNA repair and telomere maintenance were highly upregulated in keratinocytes grown on fHDF ECMs, and these genes were moderately expressed or downregulated when keratinocytes were grown on aHDF ECMs or collagen I respectively. Collectively, these data indicate keratinocytes cultured on fHDF ECMs have increased growth potential, which is in accordance with the data in figure 4.

### 2.5. fHDFs and aHDFs contain distinct subpopulation of cells

The aHDFs and fHDFs were re-examined to investigate if they contained cell subpopulations as reported by others. Rinkevich *et al.* [14] described a subset of dermal fibroblasts defined by their CD26 expression, which contributed to scar formation in adult skin. We also examined our aHDFs and fHDFs for their expression of CD26.

**Figure 6.**
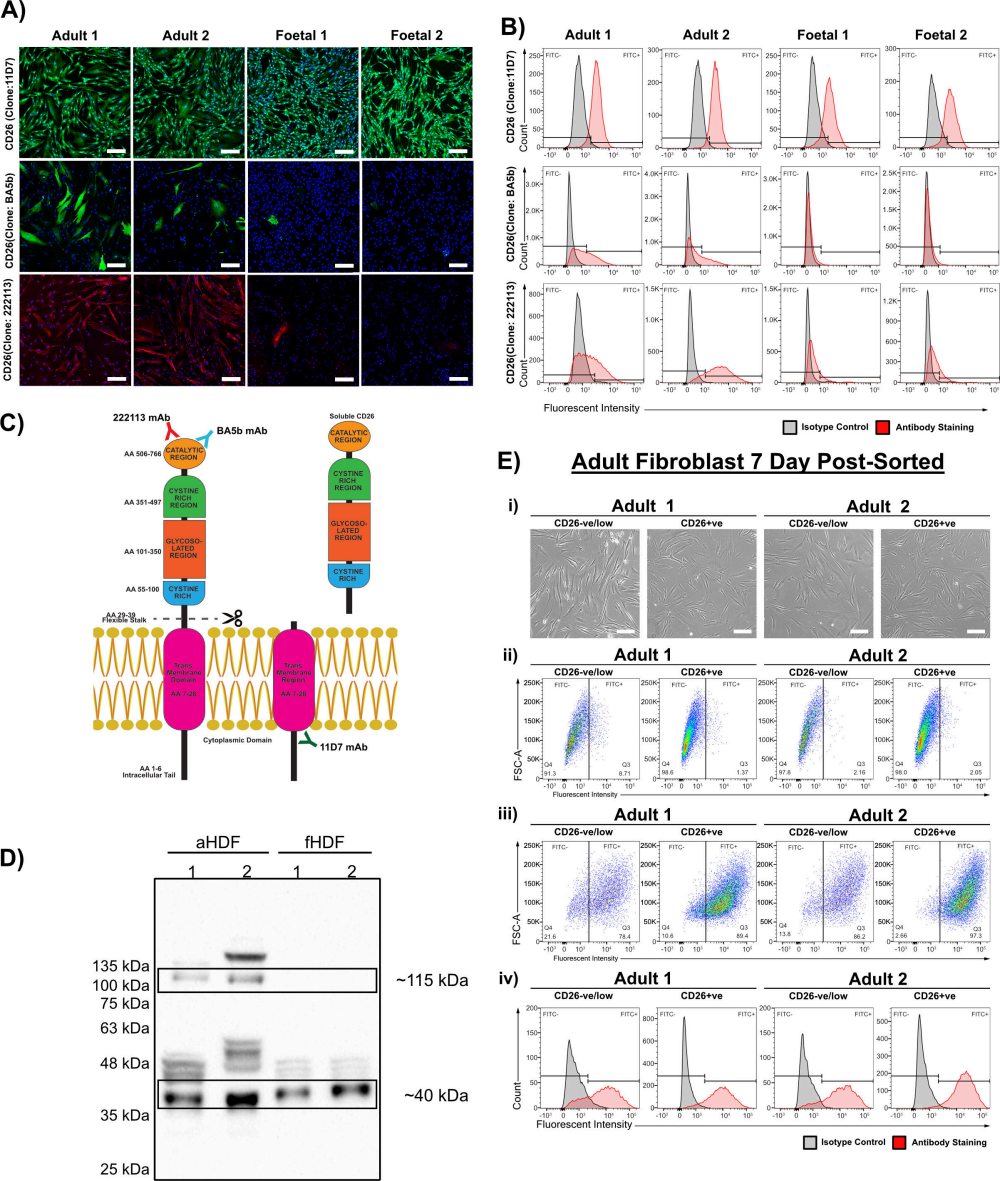
CD26 expression by adult and fetal dermal fibroblasts. (A) Immunofluorescence staining of permeabilized aHDFs and fHDFs with antibodies recognizing different CD26 epitopes. Secondary antibodies were either Alexa Fluor 546-conjugated or Alexa Fluor 488-conjugated. Nuclei were stained with DAPI (blue). Scale bars are 200 µm. (B) Histograms of FACS analyses of aHDFs and fHDFs with the different anti-CD26 mAbs. Cells stained with the mAb 11D7 were fixed and permeabilized before staining, while cells stained with the mAbs BA5b or 222113 were fixed, but not permeabilized. (C) Schematic of uncleaved and cleaved CD26. Shown are the binding regions of the mAbs used. Red = 222113 mAb; blue = BA5 b mAb; green = 11D7 mAb. (D) Western blot of aHDF and fHDF lysates resolved by gradient SDS-PAGE. Blots were probed with mAb 11D7. Molecular weights of two prominent bands are given. (E) CD26+ and CD26−ve/low subpopulations sorted from aHDFs. Data are cells sorted based on their surface binding of mAb BA5b and then cultured for 7 days before analysis again by FACS. (i) Phase contrast images of the sorted CD26+/−ve subpopulations. Scale bars: 200 µm. (ii) FACS dot plots of non-permeabilized fibroblasts immunostained with the isotype control antibody. (iii) FACS dot plots of non-permeabilized fibroblasts immunostained with CD26 (BA5b) antibody. (iv) Histograms of these data.

An initial immunofluorescence examination using the anti-CD26 monoclonal antibody (mAb) 11D7 and a cell permeabilization protocol indicated both aHDF and fHDF populations stained positively (figure 6A), which contrasted with a report indicating fHDFs had low/no CD26 expression [15]. However, other mAbs recognizing CD26 (clones BA5b and 222113) stained cells in the two aHDF populations almost exclusively. These studies were confirmed by flow cytometry (figure 6B). As the extracellular portion of CD26 can be cleaved from the cell membrane [34], it was possible the 11D7 mAb was detecting the intracellular domain of CD26 (figure 6C), while the mAbs BA5b and 222 113 bound epitopes on extracellular CD26. Re-examination of 11D7 mAb staining using non-permeabilized dermal fibroblasts failed to detect an immunofluorescence signal (electronic supplementary material, figure S4A,B), whereas non-permeabilized aHDFs stained well with the BA5b mAb, suggesting that the 11D7 mAb bound an intracellular epitope of CD26. A western blot of both aHDFs and fHDFs probed with the 11D7 mAb revealed bands at approximately 40 kDa and 115 kDa in the aHDF cell lysates, but the fHDF cell lysates only contained the small molecular weight band (figure 6D). Thus, while the aHDFs expressed both the full length and cleaved version of CD26, the fHDFs contained only cleaved CD26 lacking the extracellular domain.

To determine whether the CD26^−^ subpopulation within the aHDFs (stained with mAbs recognizing extracellular CD26) are phenotypically similar to the fHDFs, the aHDF CD26^−^ subpopulation was isolated by cell sorting after BA5b mAb staining. The CD26^+^ and CD26^−^ post-sorted subpopulations from both aHDF samples were examined after 7 days of culture. Morphologically, both CD26^+^ and CD26^−^ post-sorted subpopulations were similar, having a thick, spindle-like morphology (figure 6Ei). Flow cytometry using the BA5b mAb revealed CD26^+^ cells in the CD26^−^ post-sorted subpopulation and the ratio of CD26^+^/CD26^−^ expressing cells in both post-sorted aHDFs was similar (figure 6Eiii–iv). Plots of these cells stained with the isotype matched control antibody are shown for comparison (figure 6Eii).

**Figure 7.**
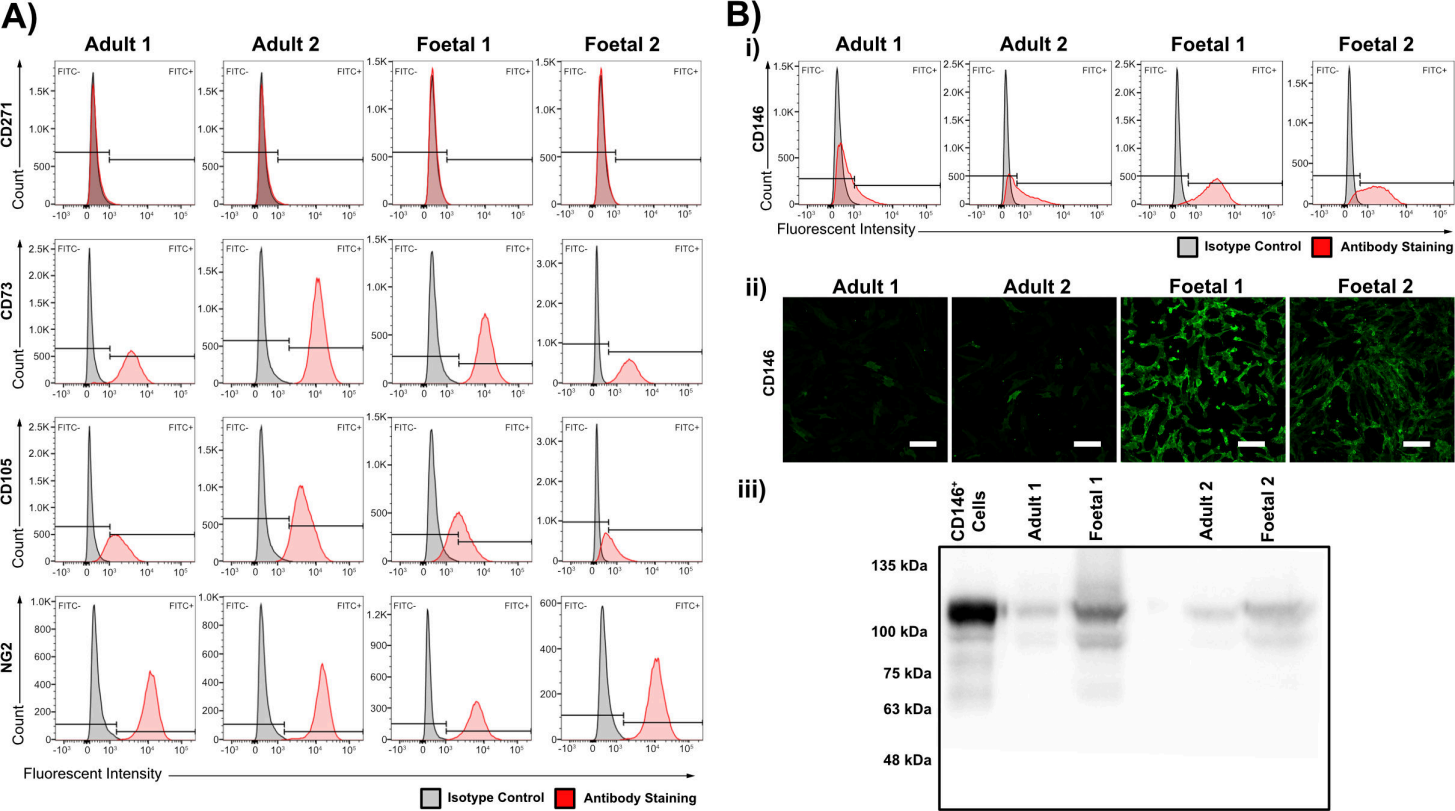
Expression of mesenchymal stem cell markers on adult and fetal dermal fibroblasts. (A) Histogram of FACS analyses of aHDFs and fHDFs with antibodies recognizing CD271, CD73, CD105 and NG2. (B) CD146 expression by aHDFs and fHDFs. (i) Histogram of FACS analysis of aHDFs and fHDFs stained with a mAb recognizing CD146. (ii) Immunofluorescence staining of aHDFs and fHDFs with the anti-CD146 mAb and an Alexa Fluor 488 conjugated secondary antibody. Scale bars = 200 µm. (iii) Western blot of cell lysates of: CD146^+^ melanoma cells, aHDFs and fHDFs resolved by SDS-PAGE and probed with an anti-CD146 mAb.

As fetal HDFs have been reported to exhibit characteristics of multipotent mesenchymal stem cells (MSCs) [35] we examined our aHDFs and fHDFs for the expression of known MSC markers (CD73, CD105, CD146, CD271 and NG2) [36]. Both aHDFs and fHDFs expressed all the markers except CD271 (figure 7A,B), although the expression levels of CD106 differed in both aHDF and both fHDF samples, reflecting sample differences for this marker. Except for the CD146 staining pattern, no clear differences in MSC marker expression were detected between the aHDF and fHDF populations. However, consistent difference in CD146 staining was observed: CD146 expression was high on most cells within the two fHDF samples, while only a small CD146^+^ subpopulation was evident in the two aHDF samples (figure 7Bi–iii).

## 3. Discussion

During epidermal development in the fetus, basal keratinocytes actively proliferate, before transitioning to a pattern of homeostatic, regulated self-renewal and differentiation as seen in adult skin. Here we showed that the ECM secreted by fHDFs direct keratinocytes to transcribe genes associated with self-renewal, whereas ECM secreted by aHDFs direct the same keratinocyte population to express genes for differentiation and proliferation. The proteomic analysis of the ECMs produced by fHDFs and aHDFs indicated these ECMs are distinct. In addition, we found that aHDFs and fHDFs comprise distinct fibroblast populations, most clearly delineated by the cleavage status of CD26 and CD146 cell surface expression but reduced Th-1 expression was also apparent on fHDFs. Moreover, our data suggested that one mechanism by which fetal dermal ECM promotes keratinocyte self-renewal is via the fine-tuning of BMP/TGFβ/SMAD signalling pathways. Accordingly, it is likely cues from the fetal dermis that direct keratinocyte self-renewal are also involved in the regenerative healing of fetal skin. Certainly, extrinsic signals from the ECM, via their regulation of keratinocyte self-renewal and differentiation, will play a fundamental role in skin development.

### 3.1. Heterogeneity in fetal and adult dermal fibroblasts

Fibroblasts from all samples were positive for vimentin and Thy-1, but less Thy-1 was expressed by both fHDF samples (figure 1A). Possibly, this is the first report of a difference in Thy-1 expression between aHDFs and fHDFs. We also observed a small but consistent subpopulation of aHDFs that lacked Thy-1 on their surfaces (figure 1C). A study of human skin sections indicated that Thy-1^+^ fibroblasts are spatially constricted, being located prominently in the lower reticular dermis, but rarely seen within the papillary dermis [37]. But, when Thy-1^−^ fibroblasts were sorted from adult skin samples, and cultured, they became Thy-1^+^ [37], indicating this marker cannot be used to differentiate whether primary fibroblasts kept in culture originated from the reticular or papillary dermis.

Our fHDFs contained a small subpopulation of myofibroblasts, consistent with the findings of Walraven *et al.* [29]. fHDFs are reported to lack a response to TGFβ1 stimulation, resulting in little myofibroblast differentiation [38]. We similarly found no change in myofibroblast differentiation following TGFβ1 stimulation of the fHDFs in standard cell culture conditions (figure 3C). By contrast, no myofibroblasts were observed in the aHDFs until they were induced to differentiate by TGFβ1 stimulation. However, when cultured under MMC conditions (the conditions used to produce our fibroblast ECMs) both aHDFs and fHDFs remained undifferentiated, regardless of TGFβ1 stimulation.

Dermal fibroblasts are not a single cell type, rather they comprise distinct subpopulations. We explored whether the different ECMs deposited by aHDFs and fHDFs were reflected by differences in the markers expressed on our fibroblasts. As CD26^+^ fibroblasts are indicated in scar formation during adult skin wound healing [14], the expression of this marker was explored. By contrast to another study [15], both our aHDFs and fHDFs expressed high levels of CD26, but the fHDF populations had cleaved CD26, where the extracellular domain was shed, while the aHDFs had both cleaved and full-length CD26 (figure 6C). MMPs cleave CD26, particularly MMP1, MMP2 and MMP14 [30]. The dominance of cleaved CD26 over full-length CD26 on our fHDFs may reflect the elevated levels of MMP2 and MMP14 we observed (figure 2). The shed extracellular region of CD26 (soluble CD26; sCD26) retains enzymatic activity in plasma and other bodily fluids. sCD26 induces activation of NF-κB signalling in HDFs [39], resulting in expression of fibrosis-associated proteins causing HDF differentiation into myofibroblasts. In culture, without MMC, the fHDFs were more prone to differentiate into myofibroblasts than the aHDFs (figure 3C). Possibly the higher levels of sCD26 in the fHDF cultures contributed to this tendency.

### 3.2. Fetal dermal fibroblast have MSC and pericyte characteristics

Reports indicate that fHDFs share MSC characteristics and the markers Thy-1, CD105 and CD73 [40]. We examined if our aHDFs and fHDFs also expressed these and other MSC markers (CD271, CD146 and NG2). CD146 had different expression levels on aHDFs and fHDFs, but NG2, CD105 and CD73 were present similarly on both fibroblast types. All our fibroblast populations lacked CD271. *In vivo* dermal CD271^+^ cells are associated with nerve fibres, and so our finding is not surprising [36]. A high proportion of CD146^+^ cells were observed within the fHDF population, suggesting the majority of fHDFs had MSC-like, or pericyte-like properties, as CD146, NG2, Thy-1 and CD73 are also pericyte markers [41]. Others reported finding CD146^+^ fibroblasts confined to perivascular locations in the papillary dermis of adult skin samples [37] but whether this is also true for fetal skin is unknown. The therapeutic potential of MSCs in wound healing is well known, particularly their ability to create a microenvironment that is permissible for tissue regeneration [42]. Similarly, pericytes can regulate keratinocyte proliferation, and dermal pericytes have been described as displaying the phenotypic and functional properties of MSCs [43].

### 3.3. Adult and fetal dermal ECMs are compositionally different

Our quantitative proteomic analysis revealed striking differences in the structural ECM proteins deposited by fHDFs or aHDFs. Higher levels of collagens I, III and IV, fibronectin, versican, agrin and biglycan were observed in the fHDFs matrices compared with the adult matrices, whereas aHDFs matrices had more decorin and collagen VI. All of these findings are in agreement with the literature [4–6,10,11,44–47]. Although fHDFs were reported to produce more collagen V [48], we observed higher levels of collagen V chains (COL5A2 and COL5A3) in the aHDFs matrices. During skin development, collagen V α1 chain transitions to an α2 chain [49]. Wenstrup *et al.* [50] showed that collagen V is essential for initiating and regulating collagen fibril assembly, possibly the differences in COL5A2 and COL5A3 levels between aHDFs and fHDFs matrices contributed to the differences in the collagen fibril structures we observed. More collagen IV and agrin were detected in the fHDFs matrices than in the adult ECM. As well as participating in basement membrane (BM) assembly [51], collagen IV is crucial for maintaining the stem cell-like characteristics of basal keratinocytes [52] and agrin is enriched in early wound microenvironments where it coordinates efficient healing [53].

Periostin, a core matrisome protein that was abundant in fHDFs matrices, is expressed in the BM at dermal–epidermal junctions during early development. As gestation progresses, its expression diminishes and it localizes around hair follicles [54]. Periostin may contribute to dermal regeneration, as it localized to the ECM during tissue remodelling in wound healing [55,56], and periostin-deficient adult mice had delayed wound closure, delayed re-epithelization and reduced keratinocyte proliferation [55]. SPARC was also abundant in fHDF matrices and, like periostin, it has been associated with dermal fibrosis, possibly because it is involved in collagen fibrillogenesis [57]. Others reported a decline in the abundance of dermal SPARC as skin ages, and the addition of SPARC to three-dimensional cultures of foreskin fibroblasts resulted in increased collagen I synthesis and decreased secretion of MMP-1 [58]. Possibly, the abundant SPARC in our fHDF ECMs contributed to the significantly thicker fHDF ECMs, as determined by collagen I immunostaining. A protein content analysis confirmed the fHDF matrices contained more protein than the aHDF matrices, which is consistent with higher matrix protein secretion levels and reduced degradation in the fHDF cultures. However, the contribution of SPARC to this finding awaits further analysis.

Dynamic remodelling of the ECM is mediated by ECM regulators (proteinases and their inhibitors), and this remodelling is crucial for embryonic development, morphogenesis, tissue remodelling and wound healing [3,59]. The major proteinases involved in remodelling are MMPs, ADAMs, ADAMTS and their inhibitors [3,59]. We found fHDF matrices had high levels of ADAM12 and ADAM19, but the in aHDF ECMs two ADAMTS were abundant. ADAMTS2 and ADAMTS1 are secreted proteases; the former is involved in procollagen processing and is essential for the maturation of collagen I fibrils in skin, whilst the latter cleaves proteoglycans like versican and aggrecan [60]. ADAMTSs and ADAMTSL (ADAMTS-like) proteins, of which two are abundant in aHDF ECMs, also function in fibrillin microfibril formation [61]. By contrast, ADAMs are integral membrane proteins and generally act as sheddases to release the ectodomains of cell surface proteins, although ADAM12 can cleave ECM proteins like collagen IV and fibronectin [62]. Also involved in collagen maturation, and abundant in fHDF ECMs, was SERPINH1. It encodes heat shock protein 47, which acts as a molecular chaperone for collagens, and together with SPARC it ensures the correct folding of procollagen molecules [57]. Of interest are LOX and LOXL1; LOX is involved in cross-linking elastin and collagen during development, whereas LOXL1 functions primarily in elastin biogenesis in the adult [63]. This fits with our findings, as we see abundant LOX in fHDF ECMs, whilst LOXL1 was most abundant in adult ECMs.

The aHDF ECMs had abundant enzyme inhibitors, including five SERPIN family members, TIMP3 and three ITIH chains, two of which can complex with AMBP (bikunin) to form inter-alpha-trypsin inhibitors (IαI). IαI complexes inhibit hyaluronidase and interact with hyaluronan to stabilize the ECM [64]. The enzymes and inhibitors in aHDF ECMs suggest these ECMs were, undergoing structural remodelling and stabilization away from the fibroblasts, which may have contributed to the thicker collagen and fibronectin fibrils we saw in adult ECMs. The abundance in aHDF ECM of two ADAMTSs and two ADAMSTLs supports this view. The fHDF ECMs resembled a thick, dense meshwork of thin fibrils of collagens and fibronectin. Possibly the matrix modifying enzymes in fHDF ECMs and the high total protein content contributed by abundant fibronectin and collagen I, III and IV chains, contributed to restricting the matrix to a more immature state. Clearly, some fHDF matrix proteins were modified at the fibroblast surface, possibly early in fibril formation. The abundance in fHDF ECMs of MMP14 and TGM2, both of which can function in ECM assembly, are consistent with this view. MMP14 releases collagen I fibrils from cells [65] and TGM2 bound to integrins cross-links fibronectin and facilitates fibronectin fibril assembly [66].

### 3.4. Adult and fetal dermal ECMs are functionally different

Our data clearly showed that ECMs secreted by aHDFs and fHDFs directed keratinocyte behaviour. When keratinocytes were grown on an aHDF matrix, gene ontology (GO) analyses revealed upregulation of genes related to keratinocyte differentiation. Particularly striking was the upregulation of numerous genes encoding proteins associated with the cornified envelope; these included the late cornified envelope genes (*LCE1F*, *LCE1B*, *LCE1A*, *LCE6A*, *LCS2C*, *LCE3D* and *LCE3E*), periplakin (*PPL*) and cornifelin (*CNFN*). Similarly upregulated were *FLG* and *LOR*, encoding respectively profilaggrin and loricrin, which occur in terminally differentiating keratinocytes located in the granular layer of skin [1]. However, when the same keratinocyte population was cultured on an fHDF matrix, the GO analysis revealed a multitude of upregulated genes related to cell division including *CDK1*, *FOXM1*, *MYB*, the cell cycle regulator *CDC27* (cell division cycle protein 27), *NEDD1* (a centrosome protein), *SPDL1* (a protein functioning in mitotic spindle formation and segregation of chromosomes) and many others.

During early epidermal development, active cell division within the basal and suprabasal layers, enables rapid expansion of the epidermis during fetal growth [59]. Our data suggest that the composition of fHDF ECM is a key player in potentiating cell division. Upon reaching maturity, the epidermis undergoes homeostatic regulation, with epidermal renewal occurring by balancing terminal differentiation with cell division [67], a process likely supported by the different composition of aHDF ECM. Indeed, our microarray data showed expression of genes for both cell proliferation and differentiation in keratinocytes grown on an aHDF ECM. Maintenance of genomic stability during cell division is crucial for normal development and homoeostasis. DNA repair prevents the accumulation of DNA damage which could compromise genomic integrity [68]. Our microarray data indicated that both aHDF and fHDF matrices contained signals which promote the expression of genes involved in DNA repair. By contrast, genes responsible for DNA repair were downregulated in keratinocytes cultured on collagen I. This indicates that alone, collagen I, cannot elicit the full complement of signalling pathways required to regulate genomic integrity, hence collagen I is not an ideal microenvironment for keratinocyte expansion.

Following injury, differentiated epidermal cells can reacquire their ‘stemness’ and participate in regenerating skin epithelia [69] and its associated appendages [13]. Our observations suggest that keratinocytes cultured on fHDF matrix, similarly reacquire their potential for skin regeneration. Indications for this include the elevated expression of genes associated with chromatin remodelling (figure 5B), and increased *LGR5* expression (figure 5D). In skin, *LGR5* is only expressed in actively proliferating hair follicle stem cells (HFSCs) located in the hair follicle bulge region of the epidermis [70], and LGR5^+^ HFSCs contribute to the regeneration and neogenesis of hair follicles and re-epithelialization of an injured epidermis [71].

### 3.5. Fetal dermal ECM regulates self-renewal and differentiation through BMP signalling

**Figure 8.**
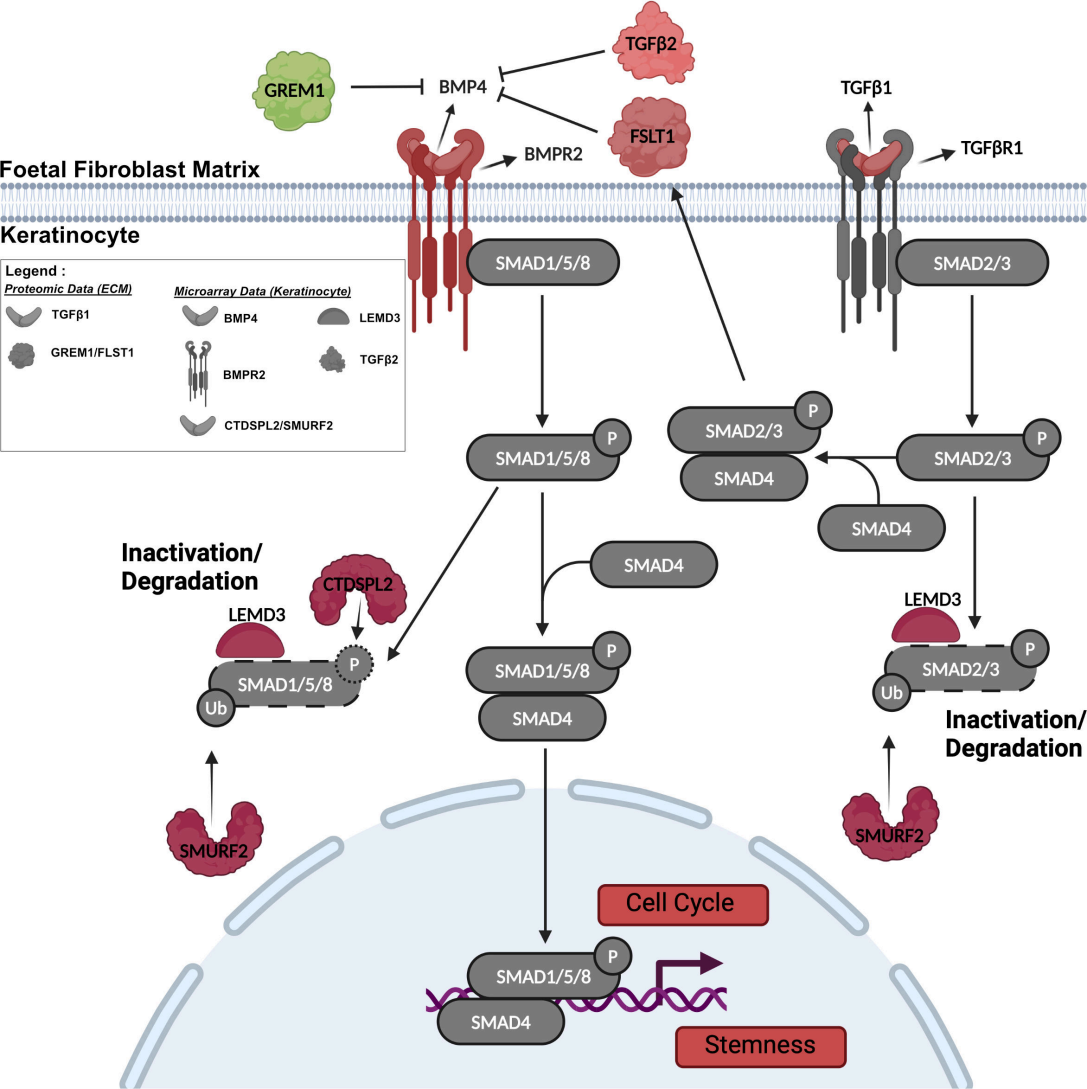
Fine-tuning of BMP4/TGFβ1 signalling by keratinocytes on fetal dermal fibroblast-derived matrix. A schematic of the BMP4/TGFβ1 signalling pathway was created using BioRender. Colours indicate upregulation (red), downregulation (green) or no change (grey) in the expression of the proteins. GREM1, FSLT1 and TGFβ2 are BMP4 antagonists found in fHDF matrices. BMP4 binds its receptor BMPR2 triggering phosphorylation of SMAD1/5/8. Phosphorylated (P) SMAD1/5/8 complexes with SMAD4, and both are transported into the nucleus to mediate BMP-dependent transcription of genes involved in the cell cycle and stemness. TGFβ1 binds its receptor TGFβR1 triggering phosphorylation of SMAD2/3. pSMAD2/3 complexes with SMAD4, which upregulates FSLT1 expression. pSMAD1/2/3/5/8 are inactivated by LEMD3 binding, and pSMAD1/5/8 are dephosphorylated by CTDSPL2. Ubiquitination (Ub) of pSMAD1/3/5 by SMURF2 results in their inactivation and degradation.

Normal tissue regeneration and homeostasis occurs by balancing quiescence and stem cell activation. BMP/TGFβ/SMAD signalling plays a key part in these processes in skin development. In skin the binding of BMPs or TGFβs to their receptors causes phosphorylation of SMAD1/5/8 or SMAD2/3 which then form distinct, two-component transcription factors with SMAD4 (figure 8) [72,73]. *In vitro* BMP4, in conjunction with retinoic acid, can differentiate human pluripotent stem cells into keratinocyte-like cells [74] and epidermal WNT activates a BMP4-FGF signalling cascade to orchestrate basal keratinocyte proliferation during stratification of the embryonic epidermis [75]. However, over-expression of SMAD1 in the epidermis compromises skin wound healing by slowing keratinocyte proliferation and migration via a BMP mediated pathway involving BMPs 4 and 7 [76], indicating a need to regulate BMP signalling to ensure normal epidermal function.

Our data support BMP/TGFβ/SMAD signalling as one of the mechanisms by which fHDF ECM maintains keratinocyte proliferation over premature differentiation. Microarray data showed keratinocytes cultured on fHDF ECMs had increased expression of *BMP4* and the BMP4 receptor, *BMPR2* (figure 5D). Extracellularly, direct BMP4 antagonists include GREM1 (gremlin 1), CRIM1 and FSTL1, and transcription of the latter is induced by TGFβ1 [77–79]. Our ECM proteomic data revealed lower GREM1 levels and higher levels of CRIM1 and FSTL1 in fHDF ECMs (figures 2 and 8). BMPs and TGFβs do not have a range of action further than one or two cells [77], hence their tethering to biglycan and perlecan contributes to their regulation and particularly so in fetal dermal ECM where these proteoglycans are abundant. TGFβ2 signalling can suppress BMP4 signalling in primary keratinocytes [80], and TGFβ1 stimulates keratinocyte proliferation in a wound healing model [81]. Our gene array data revealed elevated transcription of *TGFβ2* in keratinocytes cultured on fHDFs and our proteomic analysis revealed markedly more TGFβ1 was associated with fHDF ECM than with aHDF ECMs (figures 5D and 2): the latter is consistent with the literature [29]. Intracellularly, CTDSPL2 (C-terminal domain small phosphatase-like 2, or SCP4) dephosphorylates SMADS 1, 5 and 8, but not SMAD2 and SMAD3, meaning it negatively regulates BMP-SMAD signalling [82–84]. LEMD3 binds and inactivates SMADs 1, 5, 8, 2 and 3, and SMURF2 induces the ubiquitination of SMADs 1, 5 and 3, causing their degradation and inactivation, thus these factors suppress both BMP and TGFβ signalling [85]. Expression of both *SMURF2* and *CTDSPL2* was markedly higher in keratinocytes from fetal ECM and expression of *LEMD3* was a little higher in these same keratinocytes (figure 5D). These findings suggest fine-tuning of both BMP4 and TGFβ1 signalling occurred in keratinocytes cultured on fHDF matrices (figure 8). Interestingly, a study using airway epithelia revealed that inhibition of both BMP and TGFβ signalling enhanced dedifferentiation and permitted the long-term expansion of epithelial basal cells. Moreover, inhibition of BMP/TGFβ/SMAD signalling diminished terminal differentiation of basal epithelia and from these findings this group developed a protocol for the serial expansion of airway basal stem cells [86,87]. While TGFβ1 signalling is necessary for fetal skin development [29], possibly fHDF matrices provide a microenvironment that modulates both BMP4 and TGFβ1 signalling in non-fetal keratinocytes, thereby enabling keratinocyte dedifferentiation and self-renewal without differentiation as is seen in regenerative healing and in developing skin.

## 4. Conclusions

We have shown that dermal fibroblasts are essential contributors to the ECM compositional differences between adult and fetal dermis, as revealed by our high-quality proteomics data on the composition of the ECM produced by fHDFs and aHDFs. We have also shown that fetal human dermal fibroblasts express the markers CD26 and CD146. CD26 on fHDFs lacked its extracellular domain indicating proteolytic cleavage; by contrast, CD26 on aHDFs retained its extracellular domain. Finally, and most importantly, we demonstrated that compositional differences between of aHDF and fHDF ECM direct keratinocyte self-renewal and differentiation, and our data are consistent with BMP4/TGF-β1/SMAD signalling being involved in the processes by which fHDF ECM maintains keratinocyte self-renewal.

Collectively, these findings have revealed a fundamental aspect of skin development which previously has been overlooked. Key cell events such as cell proliferation, migration and differentiation are controlled by reciprocal interactions between intrinsic transcriptional regulation and extrinsic signals [88]. Our study highlights the profound effects extrinsic signals generated by dermal fibroblast ECMs have on keratinocyte gene expression. The different transcriptional profiles seen when keratinocytes were cultured on either fHDF or aHDF ECMs reveals the critical importance of cell–matrix interactions in controlling cell behaviour. Ang *et al.* [89] found placing MSCs in an ECM with a pro-adipogenic composition resulted in more efficient differentiation towards the adipocyte lineage compared with what was achieved using an ECM of a different composition. We show here the same is true for keratinocytes, placing non-fetal keratinocytes on a fetal ECM directed gene expression to that expected for keratinocytes in a developing fetus. Thus, the composition of the dermal ECM precisely controls a keratinocyte’s fate and as a consequence skin development and wound healing.

## 5. Material and methods

### 5.1. Antibodies

The primary rabbit polyclonal antibodies used were anti-collagen I (Abcam, ab34710), anti-collagen III (Abcam, ab7778) anti-collagen IV (Abcam, ab6586), anti-fibronectin (Abcam, ab2413) and anti-nidogen (Santa Cruz, sc-33141). The mouse monoclonal antibodies (mAbs) used were anti-α-smooth muscle actin (clone 1A4; Sigma, A2547), anti-versican (clone EPR12277; Abcam, ab177480), anti-CD105 (clone 43A3; BioLegend, 323202), anti-CD146 (clone CC9.c119; a gift from Paul Simmons), anti-CD26 (clone 11D7; Abcam, ab114033), anti-CD26 (clone BA5b; BioLegend, 302702), anti-CD26 (clone 222113; R&D Systems, MAB1180), anti-CD271 (clone ME20.4; BioLegend, 345101), anti-CD73 (clone AD2; BioLegend, 344002) and anti-vimentin (clone V9; Dako, M0725), anti-NG2 (clone MEL62; BioLegend, 867701). The secondary antibodies used were Alexa488 anti-mouse IgG, Alexa546 anti-mouse IgG, Alexa 488 anti-rabbit IgG, Alexa546 anti-rabbit IgG (from ThermoFisher Scientific) and HRP-conjugated anti-mouse IgG (Dako, PO447).

### 5.2. Cell cultures

Primary aHDFs originated from adult human surgical waste skin from abdominoplasties, donated for research with fully informed consent. These cells, none of which were immortalized, were obtained from ATCC (Adult 1: PCS-201-012) and the Institute of Medical Biology, Singapore (Adult 2). Adult 1 cells were from a 34-year-old female, while Adult 2 cells were from a 23-year-old male. Primary fHDFs from fetal dorsal skin samples of 12 and 13 weeks’ gestation were obtained from ATCC (Foetal 1: WS1-CRL-1502) and the Institute of Medical Biology, Singapore (Foetal 2); Foetal 1 was from a female, while Foetal 2 was from a male. All human cell samples were used with ethical approval from the relevant local ethics review committees in Perth, Australia and Singapore. Dermal fibroblasts were maintained in Dulbecco’s modified Eagle’s medium (DMEM) supplemented with 10% FBS (Serana Europe GmBH), and 10 mM HEPES, 2 mM L-glutamine and 1 mM sodium pyruvate (ThermoFisher Scientific). Dermal fibroblasts from passage 7 to 12 were used for all experiments. Human neonatal keratinocytes (ThermoFisher Scientific) were cultured on tissue culture growth surfaces coated with collagen I (Sigma) in PBS (3 µg cm^−2^) and maintained in defined keratinocyte serum-free medium (DKSFM; ThermoFisher Scientific). All cells were maintained at 37°C and 5% CO_2_ in tissue culture incubators. Keratinocytes at passage 4 or 5 were used for all experiments.

### 5.3. Generating acellular dermal fibroblast-derived extracellular matrix

Dermal fibroblasts were seeded (15 000 cells cm^−2^) and allowed to attach overnight in basal medium comprising DMEM: Ham F12 (3:1) supplemented with 2% human serum (ThermoFisher Scientific), 10 mM HEPES, 2 mM L-glutamine, 1 mM sodium pyruvate and 30 µg ml^−1^ ascorbic acid (Wako Chemical; non-MMC media). This medium was replaced with fresh medium containing 7.5 mg ml^−1^ Ficoll 70 (Sigma) and 25 mg ml^−1^ Ficoll 400 (GE Lifesciences; MMC media). Dermal fibroblasts were cultured for 6 days for ECM deposition, with the MMC media changed on alternate days. To obtain acellular ECM, cells were removed using phospholipase A_2_ (PLA_2_). Briefly, cells were washed in PBS and incubated in PLA_2_ (20 U ml^−1^) (Sigma)/50 mM Tris-HCl (pH 8)/0.15 M NaCl/1 mM MgCl_2_/1 mM CaCl_2_/0.5% sodium deoxycholate/1× EDTA-Free protease inhibitor (Roche) at 37°C for 30 min. Matrices were washed with PBS before treatment with 0.02 mg ml^−1^ DNase I (Amresco) in reaction buffer (10 mM Tris-HCl (pH 7)/2.5 mM MgCl_2_/0.5 mM CaCl_2_) at 37°C for 30 min. The resulting acellular matrices were washed with PBS before use.

### 5.4. Filter paper-based protein quantification assay

The assay developed by Minamide & Bamburg [90] was used. Briefly, acellular ECMs were solubilized using 8 M Urea/50 mM Tris-HCl pH 8.0 and scraped off the plate into microtubes. Then 8 µl of protein samples were added onto a Whatman No.1 filter paper. The filter paper was air-dried before being rinsed with absolute methanol for 20 s and again air-dried. Then the filter paper was placed in 0.5% Coomassie blue staining solution and incubated at RT for 30 min with gentle agitation on an orbital shaker. The paper was destained using Coomassie blue destaining solution (40% methanol, 10% acetic acid and 50% ddH_2_O) for 2 h with gentle agitation and air-dried. A grayscale image of the filter paper was obtained using ChemiDoc XRS+Imaging System (Bio-Rad). Using Image J, the pixel density of the samples was determined, and optical densities calculated using the equation log_10_(max intensity ÷ mean intensity). A BSA standard curve (concentration range 50 –2000 μg ml^−1^) was generated by plotting the mean absorbance of each BSA standard against its concentration. This curve was used to determine the protein concentration of the unknown samples.

### 5.5. Myofibroblast differentiation

Primary aHDFs and fHDFs in DMEM/10% FBS were seeded (1.3 × 10^5^ cells per well) onto etched glass coverslips (EGC) in 24 well tissue culture plates and allowed to adhere for 3 h. EGC were prepared as described [91]. EGC were washed once with DMEM/Ham’s F12 before being replaced with either non-MMC or MMC media. Fibroblasts were grown in respective media for 24 h before being stimulated with TGF-β1 (10 ng ml^−1^) in medium containing 2% FBS, stimulation continued for 48 h. Fibroblasts were then cultured for a further 24 h in their respective media without TGF-β1 before EGC with attached cells were processed for immunofluorescence staining.

### 5.6. Immunofluorescence staining

Cells or ECM were prepared on EGC in 24-well plates. Cells or ECM were fixed with 4% paraformaldehyde (PFA) in PBS for 15 min at RT, washed with PBS then blocked with 10% Goat Serum/1% BSA/PBS for 1 h at RT. Blocking solution was removed, and samples were incubated for 1 h at RT with primary antibody in 10% Goat Serum/1% BSA/PBS. Samples were washed 3 × 5 min with PBS before incubation for 1 h with secondary antibodies in 10% Goat Serum/1% BSA/PBS. Samples were then washed 3 × 5 min with PBS and cell samples incubated in DAPI (1 µg ml^−1^ in PBS) for 10 min. Coverslips were mounted in Vectashield antifade mounting medium (Vector Laboratories) and sealed with nail varnish. Images were captured with a Nikon A1+Confocal Microscope (Nikon). All antibodies were titrated to determine their appropriate concentration for use in experiments. To generate a 3D representation of the matrix, Z-stacked images of collagen I or collagen IV antibody-stained ECM were obtained using a Nikon A1+Confocal Microscope and images were merged using NIS-Elements AR analysis software.

### 5.7. Flow cytometry

Dermal fibroblasts were harvested and washed with PBS then resuspended in cold 0.5% BSA/PBS. Fibroblasts were incubated with the relevant antibodies, on ice for 30 min. After incubation, they were washed with cold PBS and incubated with a secondary FITC-conjugated anti-mouse antibody (Dako), on ice for 30 min. After washing with PBS, the fibroblasts were incubated with the dead cell marker zombie-NIR (BioLegend). A final PBS wash was done, before they were resuspended in 4% PFA/PBS for fixation prior to analysis on the BD FACSCanto II (BD Biosciences). Flow data were analysed with FlowJo Ver. 10.5.2 (BD Biosciences). If fibroblast permeabilization were required, cells were fixed with 4% PFA/PBS for 15 min prior to saponin treatment, then stained with the antibodies. For CD26^+^ fibroblast sorting, the fibroblasts were resuspended in cold 0.5% BSA/PBS, after antibody staining. BD FACS Jazz (BD Bioscience) was used for cell sorting, and the sorted cells were collected in phenol red free DMEM medium, then transferred to tissue culture flasks containing DMEM/10% FBS/1% Pen-Strep.

### 5.8. Dermal fibroblast proliferation

Fibroblast proliferation was measured using a CyQUANT Cell Proliferation Assay (ThermoFisher Scientific). Fibroblasts were seeded (3 × 10^3^ cells per well) into a 96 well tissue culture plate. After 48 or 72 h of culture, cells were washed twice with PBS and the plates placed in −80°C overnight. Plates were returned to RT and the CyQUANT assay solution was added to the wells and incubated at 37°C for 10 min. Fluorescence intensity was measured using a 485 nm/535 nm filter and an EnSpire Multimode Plate Reader (Perkin Elmer; MA, USA). A standard curve was generated by plotting known cell numbers against their mean absorbance values in this assay. This curve was used to determine fibroblast numbers after 48 and 72 h of culture.

### 5.9. Keratinocyte proliferation

Proliferation of keratinocytes on the substrates: aHDFs- and fHDF matrix, and collagen I (3 µg cm^−2^) was determined. Keratinocytes were seeded (1 × 10^4^ cells per well) into wells of a 48-well tissue culture plate (NUNC, ThermoFisher Scientific) containing the three substrates and grown for 3 days. Cells were fixed with 4% paraformaldehyde/PBS for 15 min at RT and incubated with PBS/1%BSA for 1  h at RT before nuclei were stained with DAPI (Sigma). Using an Olympus IX-81 high content screening inverted microscope (Olympus; Tokyo, Japan) and a 10× objective, 7 by 11 non-overlapping quadrants were imaged, to produce a 0.5  cm^2^ area image. Cell numbers (nuclei) were determined using Fiji Image J software and its ‘Find Object’ macro.

### 5.10. Keratinocyte size categorization

Keratinocytes were seeded (1 × 10^4^ cells per well) on the various substrates in the wells of a 48-well culture plate (NUNC). After 3 days of culture in DKSFM, keratinocytes were fixed with 4% paraformaldehyde/PBS for 15 min at RT, incubated in PBS/1%BSA for 1 h at RT before polymerized actin was stained with 1 unit ml^−1^ of Phalloidin-Alexa 488 (Molecular Probe) and nuclei with DAPI. Using an Olympus IX-81 high content screening inverted microscope (Olympus), 8 by 8 non-overlapping quadrants were imaged using a 20× objective lens. Cell size, as maximum outline profile or footprint, was estimated from the extent of the stained actin cytoskeleton using Cell Profiler software. Three size categories were chosen: small (cell area <2000 μm^2^), medium (cell area between 2000 μm^2^ and 4000 μm^2^) and large (cell area >4000 μm^2^).

### 5.11. Microarray analysis

After 3 days of keratinocytes being cultured on different substrates, total RNA was extracted using TRIzol (ThermoFisher Scientific). Contaminating DNA was removed by DNase I (Qiagen) treatment, and RNA was further purified using Qiagen’s RNeasy Mini Kit according to the manufacturer’s instructions. Extracted and purified RNA samples were sent to A*STAR Biopolis Shared Facility, Microarray Facility, Singapore. Before the samples were analysed by microarray, the quality of the RNA was checked using Agilent 2100 Bioanalyzer. Microarray analyses of the samples were performed using an Affymetrix HuGene 2.0 ST Chipset. The data were background corrected, normalized and analysed with R packages Limma and Oligo. Oligo is a package that allows the users to pre-process the microarray data while ‘Limma’ enables the expression of many genes to be analysed simultaneously.

### 5.12. Mass spectrometry analysis

Acellular aHDF- and fHDF-derived ECM were solubilized using 8 M Urea/50 mM Tris-HCl pH 8.0, then scraped off and transferred to a microtube. The matrix mixture was reduced with 10 mM DTT (Sigma), alkylated with 55 mM Iodoacetamide (Sigma) and diluted with 100 mM TEAB buffer to give a Urea concentration of <1 M. The matrix proteins were digested with sequencing grade endoproteinase Lys-C (Promega) and sequencing grade-modified trypsin (Promega) at a ratio of 1 : 100 at 25°C for 4 h and 18 h respectively, samples were then acidified with 1% TFA and desalted with a Sep-Pak C18 column cartridge (Water). 20 µg sample of peptide was labelled with TMT10plex isobaric tags (ThermoFisher Scientific). Labelling was quenched with 1 M Tris pH 7.5. The samples were cleaned and desalted using Sep-Pac C-18 cartridges, with elution using 10 mM Ammonia Formate. Samples were analysed using an Easy nLC 1000 liquid chromatography system (ThermoFisher Scientific) coupled to Orbitrap Fusion Mass Spectrometer (ThermoFisher Scientific). Each sample was analysed in a 60 min gradient using an Easy Spray Reverse Phase Column (50 cm × 75 µm internal diameter, C-18, 2 µm particles, ThermoFisher Scientific). Data were acquired in 3 s cycles with the parameters: MS in Orbitrap and MS/MS in ion trap with ion targets and resolutions (OT-MS 4 × E5 ions, resolution 120 K, IT-MS/MS 1000 ions/turbo scan, ‘Universal Method’).

Data analysis: The peak list (a filtered summary of key detected signals) was generated using Proteome Discoverer v. 1.4 (ThermoFisher Scientific) from raw mass spectrometry data. The MS/MS spectra were searched with the Mascot 2.5.1 (Matrix Science) search algorithm using the Human UniProt Database. The following parameters were used: precursor mass tolerance (MS) 20 ppm, IT-MS/MS 0.6 Da, 3 missed cleavages; Variable modifications: Oxidation (M), Deamidated (NQ), Acetyl N-terminal protein, Static modifications: Carbamidomethyl (C). Forward/decoy search was used for false discovery rate (FDR) estimations on peptide/PSM level, and were set at high confidence, FDR 1% and medium confidence, FDR 5%. The generated protein list was curated using the Matrisome [27] database.

### 5.13. Western blotting

Washed cell pellets were resuspended (5 × 10^6^ cells ml^−1^) in lysis buffer (PBS/1% NP-40) and incubated on ice for 30 min. Cell lysates were cleared by centrifugation and supernatants collected. Lysates were resolved using gradient SDS-PAGE and proteins were electroblotted onto a PVDF membrane. The membrane was blocked (1 h in PBS/0.1% Tween/5% skim milk) and then incubated overnight at 4°C with primary antibodies diluted in blocking buffer. After washing with PBS/0.1% Tween, the membrane was incubated with a secondary HRP conjugated antibody diluted in blocking buffer (1 h at RT). A 1 min incubation in Clarity Western ECL Blotting substrate (Bio-Rad) followed before imaging with a Bio-Rad ChemiDoc Imaging System.

### 5.14. Statistical analyses

Statistical analyses were performed using SPSS statistics software v. 22.0 (IBM Corporation). If the data were normally distributed and their variances homogeneous, a parametric test was conducted; if not, a non-parametric test was used. A *p*-value of *p* ≤ 0.05 was considered statistically significant. For normally distributed data, experiments containing two treatment data sets were analysed with a *t*‐test. For experiments containing three or more treatment datasets, one-way analysis of variance (ANOVA) was conducted followed by Tukey’s post hoc test.

## Data Availability

The Gene expression (microarray) dataset is available in GEO under the accession number GSE272016. Supplementary material is available online [92].
